# Erythema Induratum (Nodular Vasculitis) of the Lower Extremities Without Tuberculosis in Psoriasis on Secukinumab (Interleukin 17A Inhibition): A Case Report

**DOI:** 10.7759/cureus.94957

**Published:** 2025-10-20

**Authors:** Moamen Elhaddad, Sarah Hassan, Tayler Gant, Chase Tamashiro, Alexander Carrillo-Kashani

**Affiliations:** 1 Orthopaedics, Cedars-Sinai Medical Center, Los Angeles, USA; 2 Foot and Ankle Surgery, Cedars-Sinai Medical Center, Los Angeles, USA; 3 Anatomic and Clinical Pathology, Cedars-Sinai Medical Center, Los Angeles, USA

**Keywords:** erythema induratum (nodular vasculitis), interleukin-17a inhibition, psoriasis, secukinumab, tuberculosis-negative

## Abstract

Erythema induratum/nodular vasculitis (EI/NV) classically associates with tuberculosis (TB), yet TB-negative disease is increasingly recognized in low-prevalence settings. A 58-year-old nurse with palmoplantar psoriasis controlled on secukinumab developed 6-8 months of exquisitely tender, ulcerating lower-leg nodules refractory to multiple antibiotics. Cultures were largely unrevealing; TB testing and broad microbial cell-free DNA were negative. Incisional biopsies showed lobular panniculitis with mixed inflammation and focal vasculitis, consistent with EI/NV; special stains/PCR for mycobacteria were negative. Multidisciplinary management included brief targeted Gram-positive coverage, high-dose prednisone taper, temporary cessation of IL-17 blockade, and transition to colchicine for steroid-sparing control. Within weeks, no new lesions appeared, and prior ulcers healed. This case underscores the importance of clinicopathologic correlation, rigorous infectious exclusion, and coordinated dermatology - infectious diseases - rheumatology care, favoring individualized anti-inflammatory therapy over empiric antituberculous treatment when TB risk is low and diagnostics are negative.

## Introduction

Erythema induratum of Bazin (EI) is a rare form of chronic nodular panniculitis (inflammation of the subcutaneous fat) traditionally classified as a tuberculid - a cutaneous hypersensitivity reaction to *Mycobacterium tuberculosis* antigens [1]. It typically presents as recurrent crops of tender, erythematous subcutaneous nodules on the lower legs (often with ulceration) in otherwise healthy individuals, classically middle-aged women [2,3]. Histopathologically, EI is characterized by a lobular granulomatous panniculitis with accompanying vasculitis (inflammation of blood vessels) and focal necrosis [1]. Due to its historical association with tuberculosis (TB), EI is also known as Bazin’s disease, and early literature emphasized underlying TB infection as an etiologic driver [4]. However, not all patients with clinical EI have demonstrable *M. tuberculosis* in their lesions or evidence of active TB, underscoring that other factors can produce an EI-like picture. Indeed, the clinical appearance of EI can overlap with other panniculitides (e.g., nodular vasculitis, erythema nodosum) and vasculitic syndromes, making diagnosis challenging.

In modern practice, EI without any identifiable TB focus has been increasingly recognized [5]. In regions with low-TB prevalence, many cases that resemble classical EI yield negative TB testing, blurring the line between a true tuberculous hypersensitivity versus idiopathic or alternate etiologies [5]. For example, a recent 20-year study from a low-TB setting found *M. tuberculosis* DNA in only ~5% of EI biopsy specimens, leading the authors to conclude that most contemporary EI cases in that cohort were in fact TB-negative nodular vasculitis (NV) [5]. This highlights a key point: while EI was historically linked to tuberculosis, the absence of TB infection does not rule out the diagnosis. Careful clinicopathologic correlation is required, and a broad differential (including other causes of panniculitis) must be considered in such cases [1].

The term “nodular vasculitis” is often used interchangeably with EI, especially when a tuberculous cause is not found [6]. NV has been associated with both nontuberculous infections and noninfectious triggers [7]. For instance, chronic bacterial or atypical mycobacterial infections can provoke similar lobular panniculitis, and there are reports linking NV to respiratory pathogens in the absence of TB [7]. On the other hand, immune-mediated processes and even medications may incite an EI-like reaction. One illustrative case involved a psoriasis patient who developed NV during therapy with the tumor necrosis factor (TNF) inhibitor etanercept; extensive work-up in that case was negative for infections (including TB) or autoimmune disease, implicating the biologic drug as the trigger [7]. Such examples underscore the complex interplay between immune dysregulation, infection, and panniculitis in the pathogenesis of EI/NV.

Accurately distinguishing EI/NV from other conditions is critical, as misdiagnosis can lead to inappropriate treatment, such as unnecessary long-term antituberculous therapy or a delay in effective anti-inflammatory management, with significant implications for patient morbidity and quality of life.

We present an immunocompetent, TB-negative patient with biopsy-proven lobular panniculitis with focal vasculitis consistent with EI/NV, arising in the setting of long-standing psoriasis treated with an IL-17 inhibitor and accompanied by a borderline Mycoplasma IgG signal. The case underscores the multidisciplinary diagnostic pathway - dermatology, ID, and rheumatology - and the therapeutic balancing act between antibiotics and anti-inflammatory strategies when classic TB associations are absent and alternative immune or infectious contributors are plausible. This report aims to clarify a pragmatic approach to work-up and management in TB-negative EI/NV for dermatology, ID, rheumatology, and general medicine audiences.

## Case presentation

A 58-year-old female hospital-based nurse with a 10-year history of palmoplantar psoriasis, which had been in complete remission for 7-8 years on monthly secukinumab (300 mg subcutaneously), presented with a history of 8-9 months of new, recurrent, exquisitely tender nodules and ulcerative lesions on both lower legs and ankles. Her medical history included migraines, chronic sinusitis, past obesity status-post lap-band surgery, and sciatica. Her home medications at presentation, aside from secukinumab, included estradiol, medroxyprogesterone, omeprazole, and various PRN agents (e.g., methocarbamol, hydrocodone-acetaminophen). The painful leg lesions significantly impaired her quality of life, causing severe distress, sleep disruption, and an inability to ambulate or work comfortably. Lesions cycled between partial re-epithelialization and abrupt re-opening, often with purulent drainage. Pain was severe and localized to lesion sites, accompanied by chills but no documented fever. She denied trauma, injections, hot-tub/pool exposure, immersion pedicures, or other inoculation risks. Prior outpatient management involved multiple antibiotic courses (doxycycline provided transient benefit); linezolid had previously provoked a severe allergic reaction. Other reported allergies included penicillins, tetracyclines, sulfonamides, erythromycin, and morphine, in addition to shellfish.

**Figure 1:**
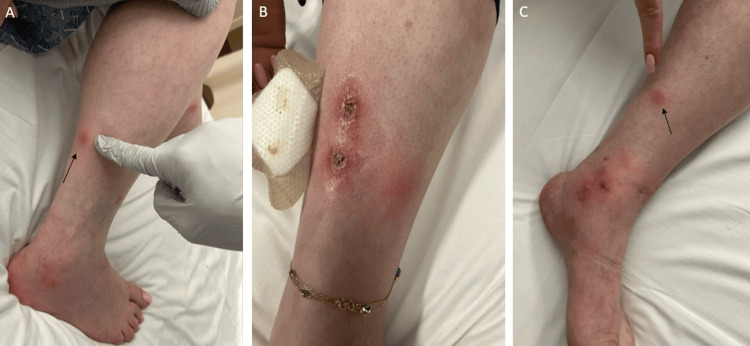
Clinical photographs of lower-extremity erythema induratum/nodular vasculitis (A) Right posterior lower leg: erythematous subcutaneous nodule (arrow), wedge-biopsied later. (B) Right anterior lower leg: two sharply marginated “punched-out” ulcers with surrounding erythema and fibrinous crust. (C) Left medial lower leg: erythematous subcutaneous nodule (arrow), wedge-biopsied later, with adjacent superficial nodular ulceration near the ankle.

On admission, she was afebrile and hemodynamically stable. Examination revealed clustered erythematous subcutaneous nodules on both legs, including sharply marginated “punched-out” ulcers on the right anterior shin with surrounding erythema and fibrinous crust (Figures 1A-1C). There were no oral or ocular lesions, synovitis, lymphangitic streaking, crepitus, or organomegaly. Initial laboratory evaluation showed systemic inflammation with leukocytosis and high acute-phase reactants; renal and hepatic function were within reference limits aside from mild hypoalbuminemia. Urinalysis was unremarkable. Serial inflammatory markers remained elevated over several days but gradually trended downward as the white blood cell count normalized. A comprehensive chronology of hematologic and chemistry data is summarized in Table 1.

**Table 1:** Summary of labs, cultures, and serologies WBC: white blood cell count; Hb: hemoglobin; Hct: hematocrit; RF: rheumatoid factor; CRP: C‑reactive protein; ESR: erythrocyte sedimentation rate; Ig: immunoglobulin; PCR: polymerase chain reaction; TB: tuberculosis; HBsAg: hepatitis B surface antigen; anti‑HBc: hepatitis B core antibody; HLA‑B27: human leukocyte antigen B27; G6PD: glucose‑6‑phosphate dehydrogenase; TPMT: thiopurine S‑methyltransferase; TSH: thyroid‑stimulating hormone; ASO: antistreptolysin O; C‑ANCA: cytoplasmic antineutrophil cytoplasmic antibody; P‑ANCA: perinuclear antineutrophil cytoplasmic antibody; PR3: proteinase 3; MPO: myeloperoxidase; RPR: rapid plasma reagin; Ag/Ab: antigen/antibody; EIA: enzyme immunoassay; ACE: angiotensin‑converting enzyme; S/CO: signal‑to‑cutoff ratio; EU: enzyme units (assay‑specific); CU: calibrated units (assay‑specific); GPL/MPL/APL: IgG/IgM/IgA phospholipid units

Test	Result	Reference Range / Units
WBC	13.90	4.00–11.00 ×10^3^/µL
Hb	13.2	11.6–15.4 g/dL
Hct	40.8	34.3–45.4 %
RF	<13	<30 IU/mL
CRP	65.2	<5.1 mg/dL
ESR	96	<30 mm/h
Mycoplasma pneumoniae IgG	0.93	≤0.90
Mycoplasma pneumoniae IgM	188	≤770 U/mL
Karius Spectrum Digital Culture (plasma)	No microbes detected at significant levels	—
Blood culture	No growth	—
Fungal culture	No growth	—
Wound culture	1+ Coagulase-negative *Staphylococcus*	—
Cyclic citrullinated peptide (CCP) IgG/IgA	<20	<20 units
IgG, quantitative (serum)	1,088	552–1,631 mg/dL
IgA, quantitative (serum)	843	65–421 mg/dL
IgM, quantitative (serum)	125	33–293 mg/dL
Immunofixation electrophoresis (serum)	Normal pattern; no monoclonal proteins detected	—
Creatine kinase (CK)	62	29–168 U/L
Mycobacterium PCR (tissue)	Not detected	—
QuantiFERON–TB Gold	Negative	—
Nil value (TB)	0.06	—
Antigen 1 – Nil (TB)	0.00	—
Antigen 2 – Nil (TB)	0.02	—
Mitogen – Nil (TB)	7.92	—
Hepatitis B surface antigen (HBsAg)	Negative	—
Hepatitis B core antibody, total (anti‑HBc)	Negative	—
Hepatitis C antibody	0.09	<0.80 S/CO
HLA‑B27	Negative	—
Glucose‑6‑phosphate dehydrogenase (G6PD), quantitative	Negative	—
TPMT enzyme activity	32.1	>18.0 EU (normal activity)
TSH	0.68	0.39–4.60 mIU/L
Anticardiolipin IgG	<10	<20 GPL
Anticardiolipin IgA	<10	<20 APL
Anticardiolipin IgM	34	<20 MPL
Antistreptolysin O (ASO) titer	146	≤330 IU/mL
Vitamin D, 1,25‑dihydroxy	55.1	19.9–79.3 pg/mL
C‑ANCA	<1:20	<1:20 (titer)
P‑ANCA	<1:20	<1:20 (titer)
Proteinase‑3 (PR3) antibody	<20.0	<20.0 CU
Myeloperoxidase (MPO) antibody	<20.0	<20.0 CU
RPR (syphilis)	Non‑reactive	—
HIV‑1 RNA PCR	Not detected	—
HIV‑1/2 Ag/Ab, 4th generation	Nonreactive	—
Coccidioides IgG, EIA	<0.150	<0.150
Coccidioides IgM, EIA	<0.150	<0.150
Cryptococcal antigen	Negative	—
Angiotensin‑converting enzyme (ACE)	54	16–85 U/L
Cryocrit	Negative	—

Imaging supported a deep cutaneous process without osteoarticular involvement. Radiographs of the tibiae/fibulae and ankles demonstrated diffuse soft-tissue swelling without osseous erosion, periosteal reaction, or soft-tissue gas. Bilateral ankle-brachial indices were normal. Focused sonography of the lower legs showed subcutaneous edema compatible with cellulitis on one side and no focal fluid collections. Transthoracic echocardiography later revealed preserved systolic function without valvular vegetations.

Microbiologic results were discordant across sites and modalities. Two sets of blood cultures remained negative. Wound cultures were variable: one ankle site grew scant coagulase-negative *Staphylococcus *(CoNS), while other samples showed no growth despite the presence of polymorphonuclear leukocytes on Gram stain. Prior outside records documented *Staphylococcus pseudintermedius*/intermedius from a lesion. Broad molecular testing with plasma microbial cell-free DNA was unrevealing, and testing for mycobacterial and fungal bloodstream infection remained negative over the observation period. Interferon-gamma release testing for TB was negative.

**Figure 2:**
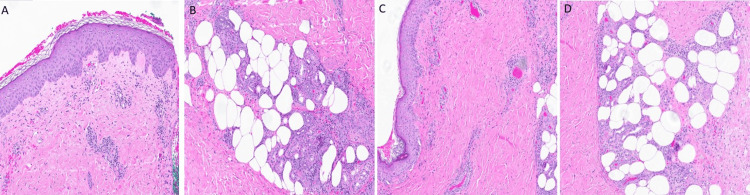
Paired lower-leg biopsies—nodular vasculitis (H&E, 8×) Right leg (A-B): Dermal and subcutaneous mixed inflammatory infiltrate with focal vasculopathic changes and mild fat necrosis, H&E, 8×, Left leg (C-D): Focal vasculitis with associated mixed lobular and septal panniculitis, characterized by fat necrosis and suppurative mixed inflammatory infiltrate, H&E, 8×.

Given the chronicity, pain severity, and limited microbiologic signal, Dermatology, ID, Rheumatology, and Podiatry were engaged early. Two incisional wedge biopsies from representative nodules (Figures 1A-1C) were obtained within the first hospital days. Histopathology demonstrated a predominantly lobular panniculitis with mixed inflammatory infiltrates and focal vasculitis, a pattern supportive of NV/EI in the appropriate clinical context (Figures 2A-2D). Special stains for bacteria, fungi, and mycobacteria were negative, and mycobacterial PCR was sent. Prior to the superficial punch biopsy several weeks earlier, a biopsy had shown granulomatous inflammation with negative stains but without diagnostic vasculitis. Taken together, the clinicopathologic picture-deep panniculitis with focal vasculitis, absence of systemic features, negative TB testing, and low epidemiologic risk-favored TB-negative NV/EI.

Empiric ceftriaxone plus vancomycin were initiated on presentation. Ceftriaxone was discontinued after several days, and vancomycin was stopped shortly thereafter once blood cultures finalized as no growth and the patient’s wounds appeared clinically quieter. During a brief antibiotic-free interval, two new nodules emerged. Given her prior severe reaction to linezolid and the mixed culture history (including the outside *S. pseudintermedius* isolate), ID recommended repeating blood cultures and then starting daptomycin to provide targeted Gram-positive coverage while minimizing marrow suppression and drug-drug interactions. A baseline creatine kinase was obtained, and a peripherally inserted central catheter (PICC) was placed to facilitate outpatient dosing.

After endocarditis was excluded, dermatology began a high-dose oral prednisone taper over three weeks to suppress the neutrophilic panniculitis component consistent with NV/EI. Rheumatology reviewed an extensive serologic and infectious work-up performed both prior to admission and during inpatient care. Autoantibodies, complement surrogates, and vasculitis-focused panels were unrevealing, and there were no extra-cutaneous features to suggest systemic vasculitis or connective tissue disease. With a purely cutaneous phenotype, Rheumatology recommended holding secukinumab temporarily and deferring long-term steroid-sparing strategy to dermatology once infectious contributors were confidently excluded. The patient was discharged home on daily daptomycin via PICC, the prednisone taper, and supportive measures, with secukinumab on hold and close follow-up arranged with Dermatology, ID, Rheumatology, and Podiatry.

Over subsequent weeks, no new nodules developed, and all prior ulcers re-epithelialized, leaving hyperpigmented scars; one small erythematous macule continued to regress. Systemic antibiotics were discontinued without recurrence. After completing a three-week prednisone taper, the patient remained lesion-free on colchicine 0.6 mg twice daily. Serial markers of inflammation improved (CRP ~65→7.6 mg/L; ESR high but declining). Repeat examinations noted livedo reticularis without synovitis or systemic features. Extensive serologies were negative; TB testing and mycobacterial studies remained negative. Psoriasis stayed quiescent off secukinumab.

## Discussion

EI was originally described as a tuberculous-associated panniculitis, but not all cases are linked to *M. tuberculosis*. The term “nodular vasculitis” was introduced in 1945 to denote EI-like lesions of nontuberculous origin [8]. Today, many authors use EI and NV interchangeably, though strictly the latter refers to TB-negative cases [6]. Our patient exemplified this variant: all TB tests (interferon-gamma release assay and tissue PCR) were negative, in line with recent studies where most EI cases in low-TB settings showed no *M. tuberculosis* DNA [5].

Distinguishing EI/NV from other panniculitides is challenging but critical. EI classically presents as recurrent crops of tender, violaceous nodules on the lower legs that often ulcerate and heal with atrophic scars [8]. Unlike systemic vasculitides, EI usually lacks systemic symptoms [6]. It can resemble erythema nodosum (EN) initially, but EN lesions are typically septal, non-ulcerative, and resolve without scarring. EN is often triggered by infections or medications (e.g., streptococcal or Mycoplasma infections) [9], whereas EI/NV is a lobular granulomatous panniculitis with vasculitis on histology [6]. In our case, the skin biopsy showed lobular panniculitis with focal vasculitis, confirming EI/NV and ruling out EN or other mimickers like cutaneous polyarteritis nodosa.

While TB remains a historic cause of EI, TB-negative cases are increasingly recognized. Diverse infectious and noninfectious triggers have been reported for NV. Atypical mycobacteria (e.g., *Mycobacterium marinum*) [6], bacteria such as Nocardia and Pseudomonas, and even respiratory pathogens like Chlamydophila pneumoniae have been linked to EI-like lesions [10]. Viral hepatitis and other organisms are also implicated [11,12]. Noninfectious associations with EI/NV include superficial thrombophlebitis, hypothyroidism, chronic lymphocytic leukemia, rheumatoid arthritis, Crohn disease, and systemic lupus erythematosus [11,13]. Drug-induced NV is rare, largely limited to single-case reports; propylthiouracil has been implicated [14]. Biologics may also precipitate NV - for example, biopsy-proven NV after one year of etanercept (a TNF-α inhibitor) in a psoriasis patient, improving after withdrawal [7]. Our patients’ use of secukinumab (an IL-17A inhibitor) raises a similar concern, as biologics have been known to induce cutaneous vasculitic reactions in rare cases [15]. Notably, a borderline Mycoplasma pneumoniae serology in this case hints at a possible post-infectious trigger, consistent with reports of NV following respiratory infections [10].

Management of TB-negative EI/NV requires a multidisciplinary, case-by-case approach. In our patient, dermatology, ID, and rheumatology teams collaborated to investigate and treat all potential causes. Despite extensive evaluation (cultures, broad-range PCR, and autoimmune panels), no infectious agent or systemic disease was identified. This reflects that only a small fraction of EI lesions actually harbor *M. tuberculosis*, yet a thorough work-up is essential to exclude occult TB or alternative etiologies [5]. Once active TB was ruled out, therapy focused on controlling both infection and inflammation. We provided targeted antibiotic coverage (daptomycin) for a prior Staphylococcal isolate, while simultaneously starting high-dose corticosteroids to suppress the neutrophilic panniculitis - a standard approach in idiopathic NV [7]. The patient’s condition improved rapidly: no new nodules formed, and existing ones began to heal. This mirrors reports where discontinuing an inciting drug and administering corticosteroids led to remission of NV [7]. We also held secukinumab to remove a potential trigger and reduce immunosuppression overlap.

For long-term relapse prevention, a steroid-sparing strategy was implemented. We initiated colchicine as maintenance therapy given its anti-neutrophilic properties. Although robust trial data are lacking, other therapies such as potassium iodide, dapsone, gold salts, and doxycycline have shown anecdotal benefit in chronic EI/NV [8]. Our patient’s regimen - combining targeted antimicrobials, systemic corticosteroids, and prophylactic anti-inflammatory treatment - illustrates a pragmatic management approach tailored to TB-negative EI/NV. Beyond regimen selection, the patient’s clinical trajectory is itself instructive. The steroid-responsive, antibiotic-independent trajectory with durable control on colchicine supports an immune-mediated, TB-negative NV/EI phenotype. The temporal improvement after de-challenge of IL-17A inhibition raises the possibility of a biologic-associated vasculitic reaction, recognized with other agents, though causality cannot be established without rechallenge.

## Conclusions

TB-negative EI/NV should be approached through clinicopathologic confirmation and systematic exclusion of infection rather than defaulting to antituberculous therapy. In suitable patients, particularly in low-TB regions, holding biologic therapy, initiating a corticosteroid taper, and introducing a steroid-sparing agent such as colchicine can achieve rapid control. Brief, targeted antibiotics are best reserved for documented pathogens or compelling clinical suspicion, ideally within a multidisciplinary framework.
